# Identification and validation of biomarkers in membranous nephropathy and pan-cancer analysis

**DOI:** 10.3389/fimmu.2024.1302909

**Published:** 2024-05-23

**Authors:** Yue Yang, Gu-ming Zou, Xian-sen Wei, Zheng Zhang, Li Zhuo, Qian-qian Xu, Wen-ge Li

**Affiliations:** Department of Nephrology, China-Japan Friendship Hospital, Beijing, China

**Keywords:** membranous nephropathy, pan-cancer, differential expression gene, potential link, biomarkers

## Abstract

**Background:**

Membranous nephropathy (MN) is an autoimmune disease and represents the most prevalent type of renal pathology in adult patients afflicted with nephrotic syndrome. Despite substantial evidence suggesting a possible link between MN and cancer, the precise underlying mechanisms remain elusive.

**Methods:**

In this study, we acquired and integrated two MN datasets (comprising a single-cell dataset and a bulk RNA-seq dataset) from the Gene Expression Omnibus database for differential expression gene (DEG) analysis, hub genes were obtained by LASSO and random forest algorithms, the diagnostic ability of hub genes was assessed using ROC curves, and the degree of immune cell infiltration was evaluated using the ssGSEA function. Concurrently, we gathered pan-cancer-related genes from the TCGA and GTEx databases, to analyze the expression, mutation status, drug sensitivity and prognosis of hub genes in pan-cancer.

**Results:**

We conducted intersections between the set of 318 senescence-related genes and the 366 DEGs, resulting in the identification of 13 senescence-related DEGs. Afterwards, we meticulously analyzed these genes using the LASSO and random forest algorithms, which ultimately led to the discovery of six hub genes through intersection (*PIK3R1*, *CCND1*, *TERF2IP*, *SLC25A4*, *CAPN2*, and *TXN*). ROC curves suggest that these hub genes have good recognition of MN. After performing correlation analysis, examining immune infiltration, and conducting a comprehensive pan-cancer investigation, we validated these six hub genes through immunohistochemical analysis using human renal biopsy tissues. The pan-cancer analysis notably accentuates the robust association between these hub genes and the prognoses of individuals afflicted by diverse cancer types, further underscoring the importance of mutations within these hub genes across various cancers.

**Conclusion:**

This evidence indicates that these genes could potentially play a pivotal role as a critical link connecting MN and cancer. As a result, they may hold promise as valuable targets for intervention in cases of both MN and cancer.

## 1 Introduction

Membranous nephropathy (MN) encompasses a spectrum of disorders distinguished by the thickening of the glomerular basement membrane (GBM) and the accumulation of immune complexes beneath the epithelial cells on the outer surface of the GBM (1). MN can be categorized into primary MN and secondary MN based on its underlying causes. The causative factors for MN are multifaceted, encompassing infections, autoimmune diseases, malignancies, pharmaceutical agents, heavy metals, and more.

For decades, a connection between MN and cancers has been established (2), tracing back to 1966 when Lee et al. (3) disclosed that 11% of nephrotic syndrome patients had carcinoma, the solid tumors most commonly associated with MN are lung and gastric cancers, followed by renal cell carcinoma, prostate cancer, and thymoma (4), these glomerular lesions are thought to be paraneoplastic. Nonetheless, the precise prevalence of cancer among MN patients remains elusive, with estimates ranging from 5% to 22%. A meta-analysis indicated that the cancer prevalence in MN patients was approximately 10% (5). This association is frequently observed in patients aged over 60, and most cancer cases are identified either before or concurrent with the diagnosis of MN (6), unfortunately, in most cases, the exact pathogenesis is unclear.

Microarray-based gene expression profiling is a widely utilized, high-throughput technique for investigating complex disease mechanisms (7). This approach has facilitated the identification of diagnostic and prognostic biomarkers, disease classification, monitoring of treatment responses, and understanding of disease pathogenesis (8, 9). Recently, numerous bioinformatics studies have aimed to elucidate the pathogenesis of membranous nephropathy. These studies have included searching for biomarkers (10, 11), examining their association with immune infiltration (12), identifying hub genes involved in disease mechanisms (13), and investigating the miRNA-mRNA regulatory networks related to podocyte autophagy, lipid metabolism, and renal fibrosis (14). Such bioinformatics analyses enhance our understanding of membranous nephropathy, allow for personalized molecular assessments of patients, and identify potential therapeutic targets.

Among various types of cancers, solid cancers originating from the lung, prostate, gastrointestinal tract, and breast, as well as certain hematological cancers, exhibit a closer relationship to MN (5). Some researchers have suggested that T-cell responses, especially those triggered by tumor antigens, may play a significant role in the interaction between cancers and MN (15), but the precise mechanism underlying the occurrence of cancer-associated MN remains unidentified (16). It is worth highlighting that the latest Kidney Disease: Improving Global Outcomes (KDIGO) clinical practice guidelines (17) recommend the use of the monoclonal antibody rituximab, originally employed in lymphoma treatment, as a 1B recommendation for intermediate-to-high-risk MN treatment. Furthermore, newer monoclonal antibodies, such as ocrelizumab, obinutuzumab, and ofatumumab have also been explored for MN treatment (18). As antineoplastic agents assume an increasingly pivotal role in treating immune-mediated non-neoplastic conditions (19, 20), the relationship between immune-related disorders and cancer has garnered more attention. We contend that numerous potential connections between MN and cancer remain undisclosed. Our study aims to employ bioinformatics methods to analyze the potential link between MN and pan-cancer.

## 2 Methods

### 2.1 Data source

We conducted a search in the Gene Expression Omnibus (GEO) for gene expression datasets. Firstly, we searched for “membranous nephropathy” as the keyword, selecting “series” for Entry type and “Homo sapiens” for Organisms, and then filtered the single-cell RNA sequencing dataset and bulk RNA sequencing dataset from these datasets. In cases where multiple datasets met the aforementioned criteria, we opted for the dataset with a larger sample size and a greater count of differentially expressed genes (DEGs). In addition, RNA sequencing and clinical data for 33 distinct cancer types were obtained from The Cancer Genome Atlas (TCGA).

### 2.2 Data processing of the single-cell RNA-seq dataset

The single-cell RNA-seq data underwent filtering and analysis using the Seurat R package. The filtering criteria were established with nFeature_RNA falling within the range of 300 to 7500. To mitigate batch effects among samples, the Harmony R package was employed. The ScaleData function was utilized for data normalization, ensuring zero-centered data for principal component analysis (PCA). For data dimensionality reduction, the RunUMAP function was applied, and the FindAllMarkers function was employed to identify DEGs across distinct clusters. Clustering was carried out at a resolution of 0.8.

### 2.3 DEG screening

Quantile normalization was conducted using the preprocessCore R package, and DEGs were identified through the utilization of the limma R package. Genes with an adjusted p-value < 0.05 were exclusively considered. The upregulated and downregulated DEGs from the two datasets were subjected to an intersection process to derive the shared upregulated and downregulated DEGs.

### 2.4 Enrichment analyses and senescence-associated DEGs

We conducted enrichment analyses utilizing the clusterProfiler R package for both Gene Ontology (GO) and Kyoto Encyclopedia of Genes and Genomes (KEGG) analyses. A total of 318 genes associated with senescence were identified by utilizing the cellular senescence pathway (Homo sapiens) within the KEGG database (https://www.kegg.jp/pathway/hsa04218) and the human gene set REACTOME_CELLULAR_SENESCENCE from the REACTOME database (https://gsea-msigdb.org/gsea/msigdb/cards/REACTOME_CELLULAR_SENESCENCE). The intersection between DEGs and senescence-related genes was considered as the set of senescence-related DEGs.

### 2.5 Protein-protein interaction networks

Constructing protein-protein interaction (PPI) networks for the 13 senescence-related DEGs were constructed using the STRING Database (https://string-db.org/).

### 2.6 Machine learning and identification of hub genes

we utilized least absolute shrinkage and selection operator (LASSO) regression and random forest to further refine the selection of DEGs. The kernlab R package was employed for LASSO regression, while the randomForest R package was utilized for the random forest algorithm. Subsequently, by intersecting the gene sets derived from LASSO regression and random forest, we identified the central hub genes.

### 2.7 Characterization and functional analyses of hub genes

We generated receiver operating characteristic (ROC) curves for the hub genes using the pROC R package. Correlation analyses among the hub genes were conducted using the circlize R package. To assess immune cell infiltration, we employed the ssGSEA function from the GSVA R package. Visualization of immune cell correlations, expression differences in immune cells across different groups, and correlations between hub genes and immune cells was achieved using the ggplot2 R package. Furthermore, leveraging the top 50 genes that exhibited positive correlations with the hub genes, we conducted gene set enrichment analysis (GSEA) utilizing the Reactome database. The outcomes were presented through a ridge plot showcasing the 20 most significant pathways.

### 2.8 Prediction of upstream transcription factors and miRNAs

We utilized the Regnetwork database (https://regnetworkweb.org) to predict the upstream transcription factors (TFs) and microRNAs (miRNAs) associated with the hub genes. The resulting network was visualized using Cytoscape software.

### 2.9 mRNA differential expression analysis of hub genes in pan-cancer

mRNA sequencing data and clinical information were sourced from the TCGA database. For analysis, we included only 14 cancer types that featured more than ten pairs of cancer and normal samples. These cancer types encompassed BLCA, BRCA, COAD, ESCA, HNSC, KICH, KIRC, KIRP, LIHC, LUAD, LUSC, PRAD, STAD, and THCA. The mRNA expression values from TCGA are presented as normalized RSEM values. The fold change was computed as the ratio of the mean expression in cancer samples to the mean expression in normal samples. To determine statistical significance, a t-test was employed, and the resulting p-values were adjusted using the false discovery rate (FDR).

### 2.10 Survival analysis of hub genes in pan-caner

The mRNA expression data of hub genes and the corresponding clinical survival data across 33 cancer types were integrated for survival analysis. The cancer samples were categorized into high and low expression groups based on the median gene RSEM value. The survival R package was employed to model the survival time and status for these two groups, encompassing disease-free interval (DFI), disease-specific survival (DSS), overall survival (OS), and disease-free survival (DFS).

### 2.11 Pan-cancer mutations of hub genes

Copy number variation (CNV) data were collected from the TCGA database across 33 different cancer types. CNV is classified into homozygous and heterozygous types, which include amplifications and deletions. These variations indicate the presence of CNVs on either one or both chromosomes. The frequency (percentage) of single nucleotide variation (SNV) mutations within each gene’s coding region was calculated using the following formula: Number of Mutated Samples/Number of Cancer Samples. An SNV oncoplot was generated using the maftools package. Additionally, CNV profiles were examined, and the correlation between CNV and mRNA expression was assessed. Statistical significance was determined using p-values adjusted through the FDR correction. The relationship between paired mRNA expression and methylation levels was evaluated using Pearson’s product-moment correlation coefficient, followed by a t-distribution test. The resulting p-values were then adjusted using FDR correction. Genes with an adjusted FDR of 0.05 or lower were retained for further analysis.

### 2.12 Drug sensitivity analysis

The small molecules were sourced from the Genomics of Drug Sensitivity in Cancer (GDSC) and Cancer Therapeutics Response Portal (CTRP) databases. The Spearman correlation coefficient was employed to indicate the potential correlation between gene expression and drug sensitivity. A positive correlation suggests that genes with high expression levels confer resistance to a drug, while a negative correlation suggests that genes with high expression levels render sensitivity to a drug.

### 2.13 Validation by immunohistochemistry

The experimental procedures conducted in this study were approved by the Ethics Committee of China-Japan Friendship Hospital (Ethics Approval Number: 2021–75-K43). For the control group, normal kidney tissue adjacent to the cancerous tissue removed during kidney cancer surgery was selected. All patients in the MN group were confirmed through pathological examination.

Immunohistochemistry was employed to validate the protein expression levels of the hub genes. Paraffin-embedded tissues were re-sectioned, underwent antigen retrieval, and were then treated with 5% serum to prevent nonspecific binding. Endogenous peroxidase activity was also blocked. Primary antibodies (*PI3KR1*, *CAPN2*, *TERF2IP* from Santa Cruz Biotechnology; *CCND1*, *TXN* from Cell Signaling Technology; *SLC25A4* from Affinity Biosciences) were applied and incubated at 4°C to evaluate their expression in kidney tissue. Subsequently, secondary antibodies were used, followed by standard incubation, staining, and observation procedures.

## 3 Results

### 3.1 Selecting of MN dataset

According to the search conditions, we finally screened and identified a single-cell RNA-seq dataset (GSE171458) and a bulk RNA-seq dataset (GSE108109) for analysis, and a search flow (**Supplementary Figure 1**) has been drawn up.

### 3.2 Processing of single-cell RNA-seq data

The single-cell RNA-seq dataset GSE171458 comprises 6 MN patients and 2 healthy subjects (**Supplementary Figure 2**). Following filtration, a total of 25,223 genes and 14,357 cells were retained. In the MN group, there were 543 genes with up-regulated expression and 193 genes with down-regulated expression (**Supplementary Figure 3**).

### 3.3 DEG identification

**Figure 1:**
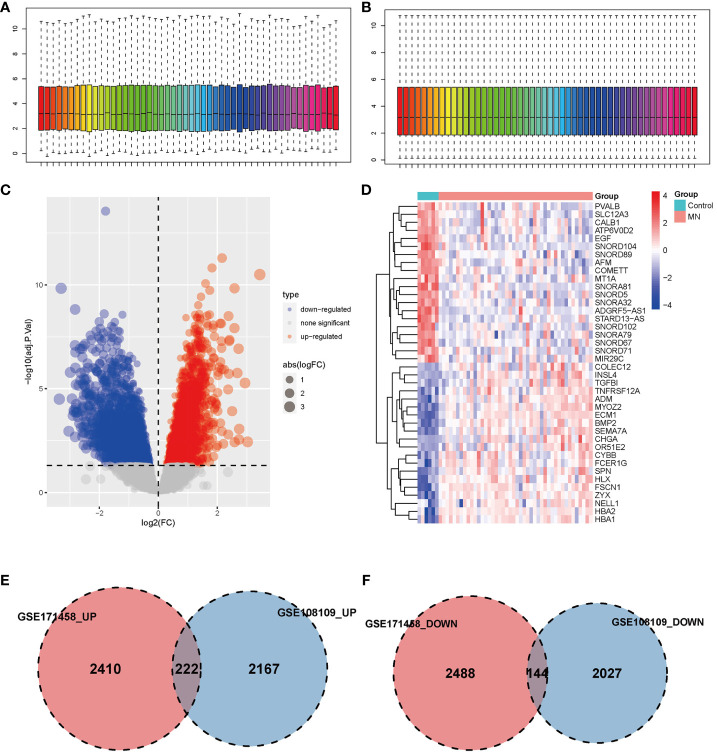
Identification of differentially expressed genes. **(A, B)** Box plot of data before and after quantile normalization from GSE108109; **(C)** Volcano plot showing the identification of upregulated and downregulated genes. **(D)** Heatmap displaying the top 20 upregulated and downregulated genes, where upregulated genes are highlighted in light red, and downregulated genes in light blue. **(E, F)** Venn diagrams illustrating the overlapping upregulated and downregulated DEGs from GSE171458 and GSE108109, revealing 222 and 144 common genes, respectively.

GSE108109 contains 44 MN patients and 6 healthy subjects, all patients with MN had a clinical presentation of nephrotic syndrome, after quantile normalization of the gene expression matrix (**Figures 1A, B**), DEGs were identified (**Figures 1C, D**). The numbers of upregulated genes in GSE171458 and GSE108109 is 2632 and 2389, respectively, while the numbers of downregulated genes were 2632 and 2171, respectively. Intersections of the up- and downregulated DEGs of the two datasets were performed separately to obtain 222 upregulated and 144 downregulated genes (**Figures 1E, F**).

### 3.4 Enrichment analyses of senescence-associated genes

To comprehensively explore the biological functions and pathways associated with the 366 common DEGs, GO and KEGG pathway enrichment analyses were conducted (**Supplementary Figure 4**). In the GO enrichment analysis, the top three biological processes exhibiting significant enrichment were cellular cation homeostasis cellular cation homeostasis (p = 5.01E-8), cellular metal ion homeostasis (p = 2.77E-7), and response to metal ion (p = 9.44E-7). In the KEGG enrichment analysis, the top three pathways were protein processing in the endoplasmic reticulum (p = 1.03E-8), amyotrophic lateral sclerosis (p = 0.000453), and Parkinson’s disease (p = 0.000102).

**Figure 2:**
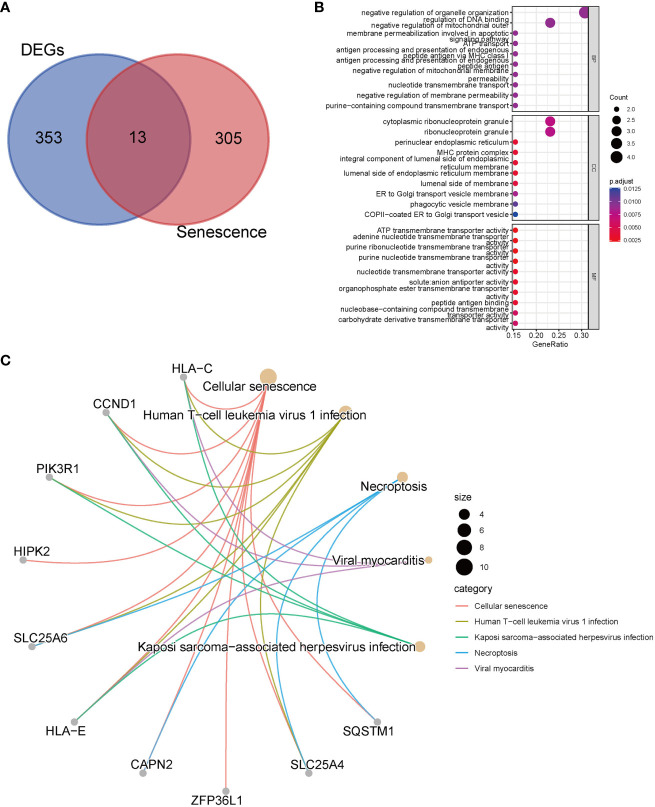
Enrichment analyses with senescence-related differentially expressed genes. **(A)** The Venn diagrams show an overlap of 13 genes; **(B)** GO analysis; **(C)** Top 5 pathways of KEGG analysis and the genes they contained.

We performed an intersection analysis between the 318 senescence-related genes and the 366 common DEGs, resulting in the identification of 13 senescence-related DEGs (**Figure 2A**). In the GO enrichment analysis, in terms of biological processes, genes exhibited significant enrichment in functions such as negative regulation of organelle organization (p = 8.61E-5), regulation of DNA binding (p = 6.98E-5), and negative regulation of mitochondrial outer membrane permeabilization involved in the apoptotic signaling pathway (p = 3.39E-5) (**Figure 2B**). The top three pathways in the KEGG enrichment analysis were cellular senescence (p = 9.64E-17), human T-cell leukemia virus 1 infection (p = 1.41E-7), and necroptosis (p = 3.85E-5) (**Figure 2C**).

### 3.5 Protein-protein interaction networks

PPI networks predictions for 13 senescence-related DEGs (**Supplementary Figure 5**).

### 3.6 Expression of senescence-related DEGs

The expression patterns of the 13 senescence-related DEGs in GSE108109 are depicted in the volcano plot and heatmap (**Supplementary Figures 6A-B**). When contrasted with healthy controls, all of the senescence-related DEGs exhibited significant expression differences, except for *TERF2IP* (**Supplementary Figure 6C**).

### 3.7 Identification of hub genes

**Figure 3:**
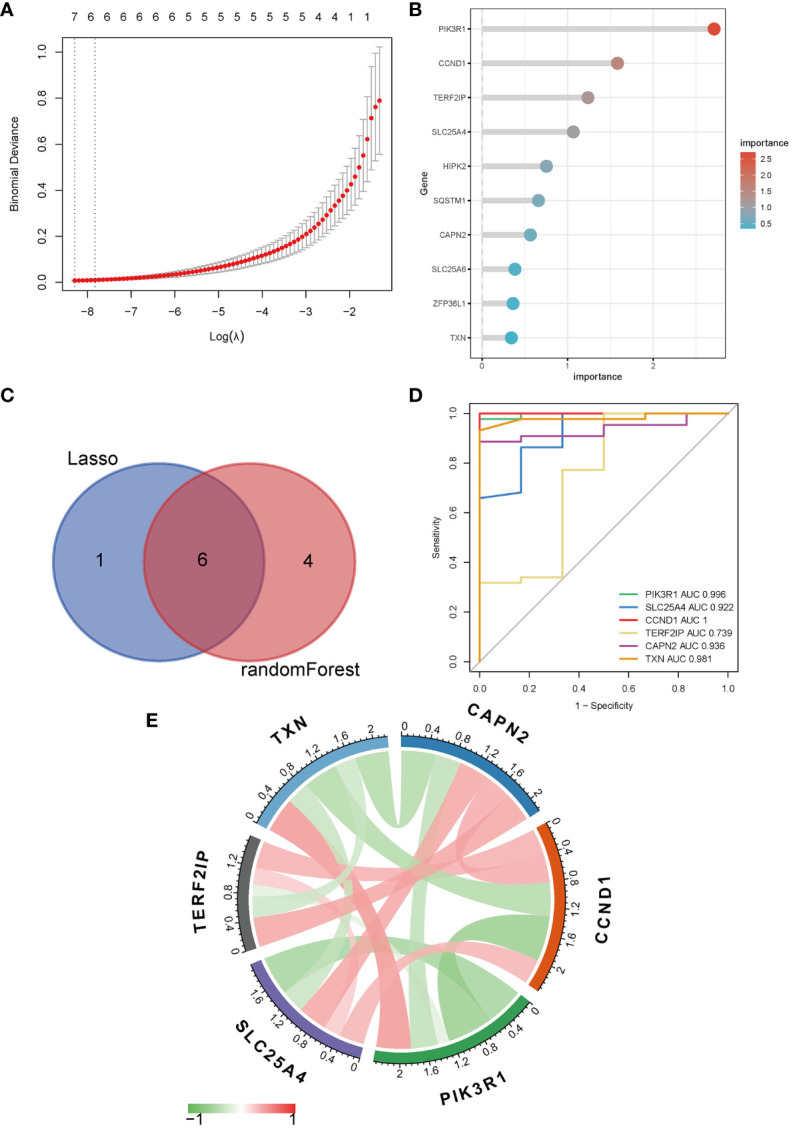
Identification of hub genes. **(A)** LASSO regression; **(B)** Top ten genes ranked by importance in random forest; **(C)** Venn diagram of LASSO regression and random forest; **(D)** ROC curve and AUC of hub genes; **(E)** Correlation analysis between hub genes, red symbolizes positive and green symbolizes negative correlation, and darker color means stronger correlation.

To further narrow down the genes, we employed machine learning techniques, namely LASSO regression and random forest. Initially, LASSO regression identified seven genes (**Figure 3A**) and subsequently, the random forest method was used to obtain the top ten genes ranked by their importance (**Figure 3B**). The intersection of these two sets yielded the final selection of six hub genes (**Figure 3C**): *PIK3R1*, *CCND1*, *TERF2IP*, *SLC25A4*, *CAPN2*, and *TXN*.

The ROC analysis revealed that the AUC values for *PIK3R1* (0.996), *SLC25A4* (0.922), *CCND1* (1.000), and *CAPN2* (0.936) exceeded 0.9, indicating a robust predictive classification capability of these hub genes for distinguishing between MN and healthy controls (**Figure 3D**). Moreover, visual representations were used to display the correlations among the hub genes (**Figure 3E**).

### 3.8 Immune infiltration of hub genes

The correlations among immune cells were intricate and widespread (**Supplementary Figure 7A**). In comparison to healthy controls, MN patients exhibited significantly elevated levels of gamma delta T cells, macrophages, mast cells, myeloid-derived suppressor cells, monocytes, natural killer cells, natural killer T cells, cytoid dendritic cells, regulatory T cells, follicular helper cells, type 1 T helper cells, and type 2 T helper cells (**Supplementary Figure 7B**). Additionally, all hub genes showed close associations with immune cell infiltration (**Supplementary Figure 7C**).

### 3.9 GSEA of hub genes

In order to gain a deeper insight into the significance of hub genes in MN and to anticipate their potential functions, we initially identified the top 50 genes in the dataset that were positively correlated with the hub genes (**Supplementary Figure 8**). Subsequently, GSEA was conducted for each individual hub gene. Focal adhesion ranked first in *CAPN2* and *CCND1*, while intrinsic component of organelle membrane, Golgi apparatus, actin cytoskeleton organization, and regulation of anatomical structure morphogenesis ranked first in *PIK3R1*, *SCL25A4*, *TERF2IP*, and *TXN*, respectively (**Supplementary Figure 9**).

### 3.10 Prediction of the upstream regulation network

**Figure 4:**
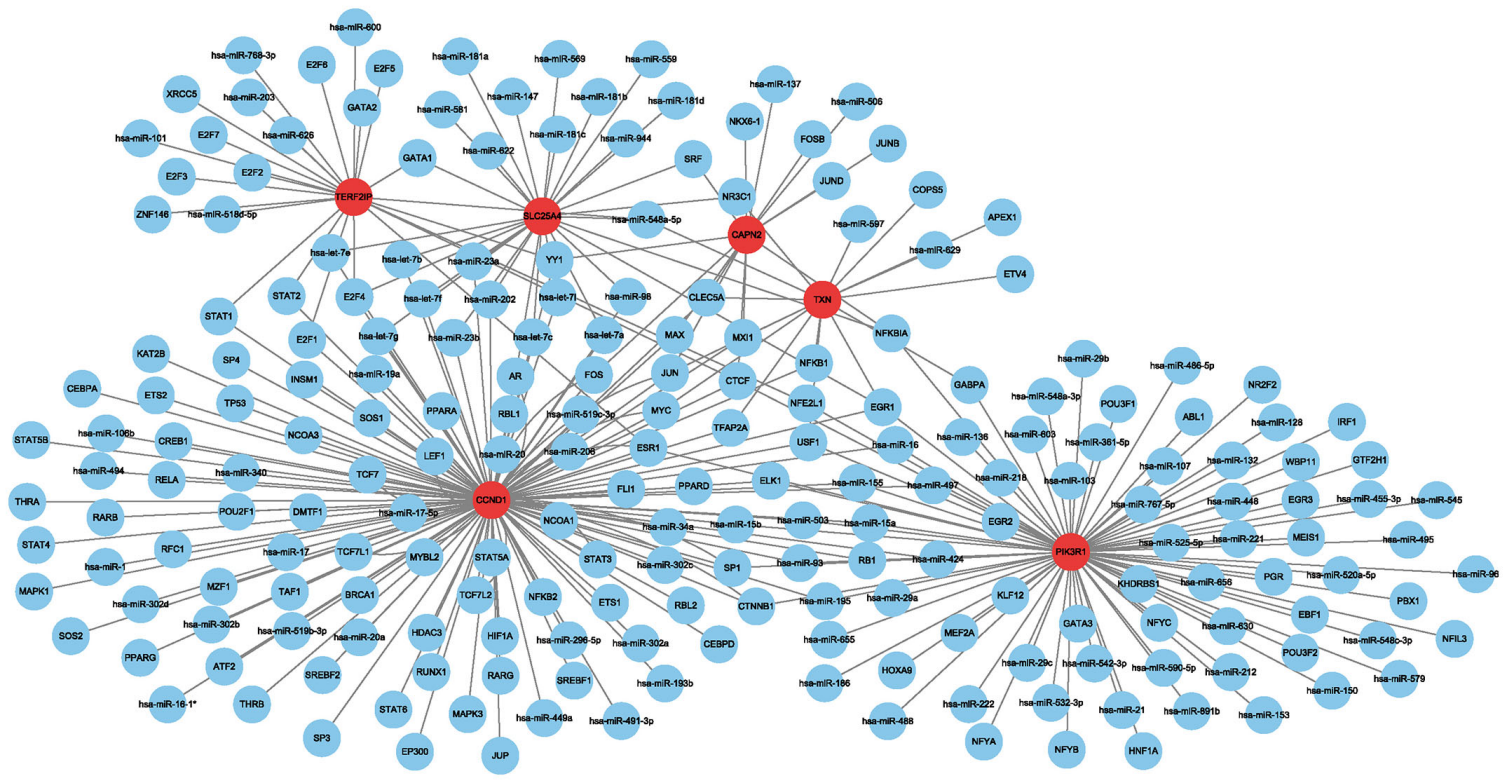
Network between transcription factors, miRNAs and hub genes. Red indicates hub genes, and blue indicates transcription factors and miRNAs.

Using the Regnetwork database, we identified TFs and miRNAs upstream of the six hub genes (**Figure 4**).

### 3.11 Hub genes expression and survival analysis of hub genes in pan-cancer

**Figure 5:**
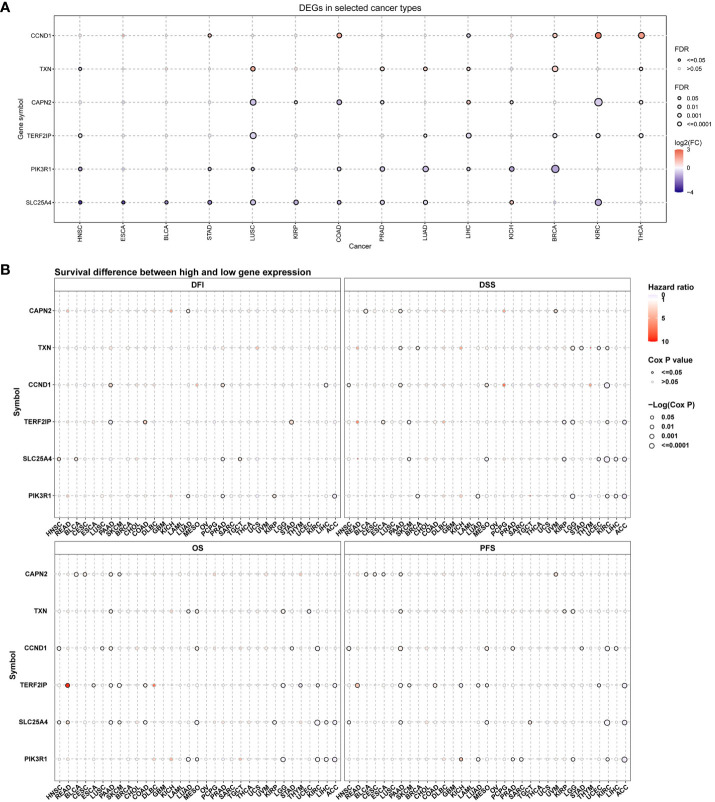
Hub gene expression and survival analysis in pan-cancer. **(A)** mRNA expression profiles of hub genes across diverse cancer types; **(B)** Assessment of the prognostic significance of hub genes in pan-cancer; DFI, progression free internal; DSS, disease specific survival; OS, overall survival; DFS, disease free survival. In this representation, red indicates elevated gene expression in cancer, blue signifies reduced expression, and a solid circle denotes statistical significance.

Hub genes were significantly differentially expressed in multiple types of cancer. Among them, *SLC25A4* was significantly differentially expressed in 11 cancers (highly expressed in KICH, and lowly expressed in HNSC, ESCA, BLCA, STAD, LUSC, KIRP, COAD, PRAD, LUAD, and KIRC), followed by *PIK3R1*, *CAPN2*, *TXN*, *TERF2IP* and *CCND1* in 9, 8, 7, 7, and 6 types of cancer, respectively (**Figure 5A**). The expression of hub genes correlates with the prognostic indicators (including progression-free internal survival, disease-specific survival, overall survival and disease-free survival) of many types of cancer (**Figure 5B**). In survival analysis, a Hazard Ratio (HR) less than 1 and a p-value less than 0.05 signify that heightened gene expression diminishes the risk of patient mortality and enhances survival—an advantageous outcome. Conversely, an HR exceeding 1 with a p-value under 0.05 indicates that increased gene expression elevates the hazard of patient demise and diminishes survival—a detrimental scenario. When HR equals 1 and p-value is less than 0.05, it suggests that heightened gene expression has no discernible impact on patient survival.

### 3.12 Mutations of hub genes in pan-cancer

**Figure 6:**
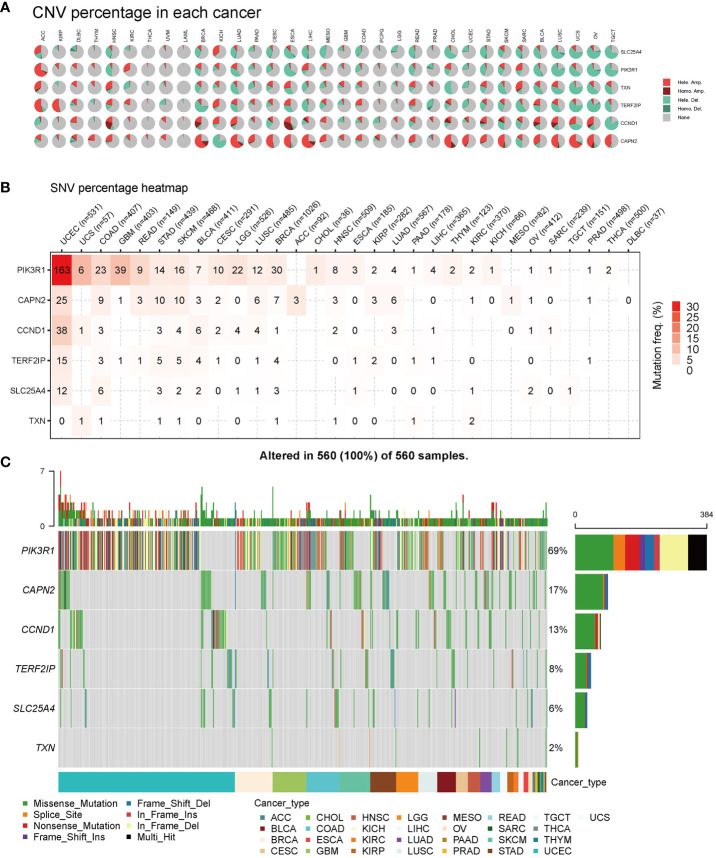
Pan-cancer mutations of hub genes. **(A)** Distribution of copy number variation (CNV) in 33 cancers. This pie chart illustrates the proportion of various CNV types for a single gene in one cancer, with different colors representing distinct CNV types. Hete Amp, heterozygous amplification; Hete Del, heterozygous deletion; Homo Amp, homozygous amplification; Homo Del, homozygous deletion; None, no CNV. **(B)** Mutation frequency of hub genes. The numbers indicate the count of samples with the corresponding mutated gene in a given cancer. ‘0’ signifies no mutation in the gene coding region, and the absence of a number indicates no mutation in any region of the gene. **(C)** Single nucleotide variation (SNV) oncoplot. This chart depicts the distribution of mutations in hub genes and categorizes SNV types.

First, we analyzed the distribution of CNV types, and the CNV pie chart revealed that the primary CNV types were heterozygous amplification and deletion (**Figure 6A**). Subsequently, we assessed the mutation frequencies of hub genes in pan-cancer, with *PIK3R1* exhibiting the highest frequency of SNV (**Figure 6B**). The mutation landscape indicated that missense mutations were the predominant type, with the hub genes displaying mutation frequencies in the following descending order: *PIK3R1*, *CAPN2*, *CCND1*, *TERD2IP*, *SLC25A4*, and *TXN*, with mutation percentages of 69%, 17%, 13%, 8%, 6%, and 2%, respectively (**Figure 6C**).

**Figure 7:**
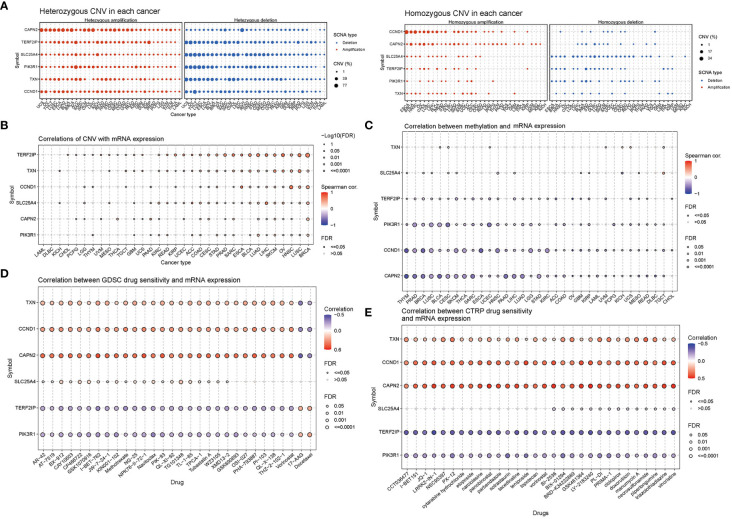
Correlation analyses of mRNA expression, CNV and drug sensitivity in pan-cancer. **(A)** Heterozygous and homozygous CNVs in pan-cancer. Genes with > 5% CNV in a given cancer are represented as data points on the figure. **(B)** Correlation between CNV and mRNA expression. **(C)** Correlation between methylation and mRNA gene expression. **(D, E)** Gene set drug resistance analysis from Genomics of Drug Sensitivity in Cancer (GDSC) and Cancer Therapeutics Response Portal (CTRP) (Top 30). The size of the data points indicates the statistical significance, with larger dots indicating higher statistical significance. The false discovery rate (FDR) was used for correction.

CNV percentage analysis revealed that hub genes displayed heterozygous amplification or deletion in nearly all cancer types, and in some cancers, they also exhibited homozygous amplification or deletion (**Figure 7A**). Correlation analysis unveiled a close relationship between the mRNA expression of hub genes and pan-cancer CNV. These genes displayed significant positive or negative correlations across multiple cancer types (**Figure 7B**). These findings suggested that CNV in hub genes mediated their aberrant expression, which could potentially play a crucial role in cancer progression. Methylation and mRNA expression correlation analysis indicated that, for the most part, the expression levels of hub genes were negatively correlated with their methylation levels, especially *PIK3R1*, *CCND1*, and *CAPN2*. Conversely, only *TXN* in BLCA, KICH, UCS, and UVM, as well as *SLC25A4* in CESC, MESO, and TGCT, exhibited a positive correlation between methylation and gene expression (P < 0.05, **Figure 7C**).

Genomic aberrations have a significant impact on the clinical response to both chemotherapy and targeted therapy treatments. To investigate the role of hub genes in chemotherapy and targeted therapy, we analyzed drug sensitivity and gene expression profiling data from cancer cell lines in GDSC and CTRP. The correlation analysis specifically refers to the relationship between gene expression and the half-maximal inhibitory concentration (IC50) of a drug, which is usually used to assess antitumor activity. A lower IC50 value indicates greater drug potency. Thus, a positive correlation implies that higher gene expression weakens the drug’s inhibitory effect, while a negative correlation suggests that higher gene expression strengthens it. The mRNA expression level of *TXN*, *CCND1*, and *CAPN2* showed a positive correlation with the sensitivity to most drugs, except for 17-AAG and docetaxel. On the other hand, the mRNA expression level of *TERF2IP* and *PIK3R1* exhibited a negative correlation with the sensitivity to most drugs in GDSC and CTRP, again with the exceptions of 17-AAG and docetaxel (**Figures 7D, E**).

### 3.13 Immunohistochemical verification of hub gene expression in the kidneys

**Figure 8:**
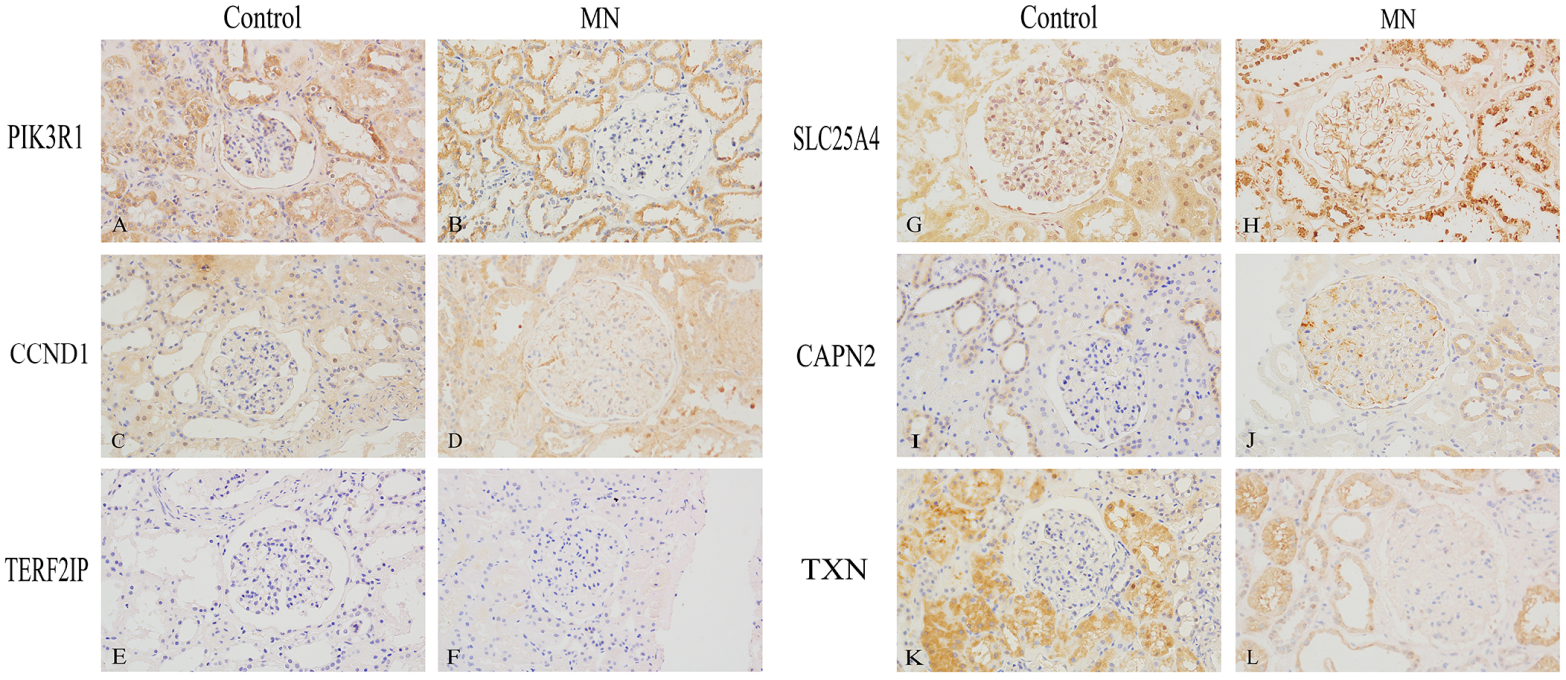
Immunohistochemical Validation of Hub Gene Expression in Human Kidney Specimens (400×). **(A, B)** *PIK3R1*, **(C, D)** *CCND1*, **(E, F)** *TERF2IP*, **(G, H)** *SLC25A4*, **(I, J)** *CAPN2*, **(K, L)** *TXN*. **(A, C, E, G, I, K)** are normal kidney tissues, while **(B, D, F, H, J, L)** are membranous nephropathy kidney tissues.

We performed immunohistochemical staining on 22 MN kidney specimens and 3 control kidney specimens (**Figure 8**). In the control group, *PIK3R1* and *SLC25A4* were expressed in both glomeruli and tubules, while *CCND1* and *TXN* were primarily expressed in tubules, with minimal expression in glomeruli. *CAPN2* was expressed in glomeruli and in some renal tubular epithelial cells. In contrast, in the MN group, *PIK3R1* exhibited reduced expression in glomeruli, while *TXN* showed significantly decreased expression in tubules. *CCND1* and *CAPN2* displayed markedly increased expression in glomerular podocytes, and *SLC25A4* exhibited increased expression along the glomerular basement membrane. *TERF2IP*, on the other hand, showed minimal expression in renal tissues in both the control and MN groups.

## 4 Discussion

In recent years, an increasing body of research has highlighted a potential correlation between immune-mediated diseases and cancer (21, 22). Possible mechanisms involved include: 1. Cancer-associated antigens induce the host to produce antibodies, and these antigens may become lodged beneath glomerular epithelial cells, forming *in situ* immune complexes that ultimately mediate renal injury (23). 2. Certain oncogenic viruses, such as hepatitis B virus, cytomegalovirus, and Epstein-Barr virus, infect the host, resulting in the production of antibodies against viral antigens. These viral antigen-antibody complexes may then deposit in the glomerulus, activate the complement system, and cause renal injury (24). 3. Necrotic tumors release substantial amounts of DNA, which triggers the body to produce anti-DNA antibodies. These immune complexes may accumulate in the kidneys, subsequently causing kidney damage (25).

In this study, we aimed to explore the potential connection between MN and pan-cancer. Our analysis led to the identification of *PIK3R1*, *CCND1*, *TERF2IP*, *SLC25A4*, *CAPN2*, and *TXN* as hub genes, which could potentially serve as targets for interventions in both MN and cancer.

The PI3K enzymes constitute a conserved family of lipid kinases, consisting of a catalytic subunit and a regulatory subunit. Phosphatidylinositol-3-kinase regulatory subunit 1 (*PIK3R1*) exhibits low expression levels in the majority of cancers and is believed to function as a cancer suppressor. Downregulation of *PIK3R1* is associated with poor survival outcomes for most cancer patients (26). Conversely, *PIK3R1* has been observed to be significantly upregulated in rats with adriamycin-induced chronic glomerulonephritis (27). Simultaneously, phospholipase A2 receptor (PLA2R), the primary target antigen in MN, has been demonstrated to activate the upregulated PI3K/AKT/mTOR pathway (28).

*CCND1* plays a pivotal role as a critical regulator of the cell cycle and holds a central position in the development of cancer by driving uncontrolled cellular proliferation. Its activity is significantly heightened in various cancer contexts (29), and the expression of *CCND1* is indispensable for the survival and proliferation of cancer cells (30). In glomerular intrinsic cells, *CCND1* was found to be expressed in podocytes in both the Heymann nephritis model of rats (31) and cases of FSGS in humans (32). It is primarily enriched in actively proliferating podocytes, as opposed to those in a quiescent state, and its expression increases following injury in passive Heymann nephritis rats (31).

*TERF2IP*, the most highly conserved component of the shelterin complex, plays a multifaceted role in the regulation of various cellular processes, encompassing cell metabolism, DNA damage response, and NF-κB signaling (33). It has been shown to play a part in oncogenesis, cancer progression, and the development of resistance to chemotherapy in human cancers, with multiple mutations and diverse expression patterns of *TERF2IP* reported in cancer contexts (34, 35). On the other hand, *TERF2IP* serves as a central signaling hub within podocytes (36). In murine disease models and kidney biopsies from glomerulosclerosis patients, injured podocytes displayed reduced activation of *TERF2IP* within the glomeruli. Notably, severe glomerulosclerosis manifests in mice with diminished podocyte expression of *TERF2IP*, leading to early mortality from renal failure by 8 weeks of age. Furthermore, podocyte-specific *TERF2IP* haploinsufficiency also resulted in significant podocyte damage, including signs of podocyte detachment (37).

The solute carrier protein 25 (SLC25) family, which is the largest gene transporter family, consists of membrane proteins that regulate the transport of various solutes in and out of cells. They play crucial roles in essential physiological processes such as cellular material transport, energy transmission, signal transduction, and nutrient metabolism. Pan-cancer analysis of the SLC25 family suggests that *SLC25A4* is linked to multiple oncogenic pathways, including the PI3K-AKT-MTOR pathway, MYC-TARGETS-V1 pathway, MYC-TARGETS-V2 pathway, and MTORC1 pathway (38). Regrettably, there have been no reported associations between *SLC25A4* and membranous nephropathy.

*CAPN2* (calpain-2) is a prototypical classical isoform of the calpain family of calcium-activated cysteine proteases. Its substrate proteins are involved in a wide range of cellular processes, including transcription, survival, proliferation, apoptosis, migration, and invasion. Dysregulated calpain activity has been linked to tumorigenesis, suggesting that calpains may hold promise as therapeutic targets (39). Interestingly, researchers have observed that inhibiting both calpain 1 and 2 in cell cycle protein G-related kinase knockout mice mitigated podocyte injury. This finding establishes a direct correlation between calpain-1/-2 activity and podocyte injury, proteinuria, and glomerulosclerosis (40).

*TXN* (thioredoxin-1) is a multifunctional protein with a molecular weight of 12 kDa, primarily localized within the cytosol. *TXN* plays a pivotal role in a diverse range of cellular functions, including cell proliferation, the maintenance of redox homeostasis, DNA synthesis, gene expression regulation, and the regulation of apoptosis-mediated cell death. *TXN* is indispensable for the normal functioning of both organs and tumors. It is strongly associated with various diseases, notably cancer, and ample evidence has been presented to underscore its significance in influencing the phenotype and prognosis of lung, gastrointestinal, and urological cancers (41). On the other hand, urinary *TXN* is regarded as a biomarker for diagnosing tubular redox dysregulation (42). Furthermore, recombinant long-acting *TXN* has been shown to ameliorate the transition from acute kidney injury to chronic kidney disease by modulating renal oxidative stress and inflammation (43).

Although a causal association between lung cancer and MN was not found in the Mendelian randomization-based study by Yang et al. (44), we don’t think they are the opposite of our conclusions. We speculate that the negative result may be related to the fact that the investigators used the primary MN dataset for their analyses. In recent years, it has become increasingly evident that the development of MN is associated with a variety of target antigens present on podocytes (45). Among the six central genes identified through bioinformatics analysis, subsequent immunostaining of renal tissues revealed a notable increase in the expression of *CCND1* and *CAPN2* within glomerular podocytes in the MN group compared to the control group. Moreover, these two genes have been substantiated to have significant implications in podocyte injury (32, 40). We believe that further exploration of these two genes in our research endeavors may unveil novel and intriguing findings in the future.

Certainly, this study has limitations. First, MN is the most prevalent pathologic type of cancer-associated nephropathy, though other types such as minimal change disease, focal segmental glomerulosclerosis, IgA nephropathy, and membranoproliferative glomerulonephritis have also been documented (5). Our study focused exclusively on MN, and thus it remains unclear whether the six hub genes identified are specific to MN. The intricate relationship between kidney disease and cancers warrants further investigation. Second, while elevated expression of *CCND1* and *CAPD2* has been noted in MN glomerular podocytes, their roles in other podocytopathies like minimal change disease are yet to be defined. Whether these genes could serve as specific markers of podocyte damage in MN or their association with MN target antigens (e.g., PLA2R, NELL1, THSD7A) require more extensive research. Third, *SLC25A4*, and *TXN* have not been previously reported in the context of MN or podocyte injury. Investigating the mechanisms underlying their roles in MN requires more research.

## 5 Conclusions

In conclusion, we identified *PIK3R1*, *CCND1*, *TERF2IP*, *SLC25A4*, *CAPN2*, and *TXN* as potential markers associated with both cancer and MN. This discovery enhances our understanding of the potential connection between cancers and MN. Furthermore, these genes represent potential therapeutic targets for MN as well as various types of cancers.

## Data availability statement

Publicly available datasets were analyzed in this study. This data can be found here: Gene Expression Omnibus at https://www.ncbi.nlm.nih.gov/geo/, reference number: GSE171458 and GSE108109.

## Ethics statement

The studies involving humans were approved by the Ethics Committee of China-Japan Friendship Hospital (Ethics Approval Number: 2021-75-K43). The studies were conducted in accordance with the local legislation and institutional requirements. The participants provided their written informed consent to participate in this study.

## Author contributions

YY: Data curation, Methodology, Validation, Writing – original draft. GZ: Methodology, Validation, Writing – original draft. XW: Funding acquisition, Methodology, Software, Writing – original draft. ZZ: Data curation, Methodology, Writing – original draft. LZ: Writing – original draft. QX: Writing – review & editing. WL: Funding acquisition, Project administration, Writing – review & editing.
