# Supplementary material for: Identification and validation of biomarkers in membranous nephropathy and pan-cancer analysis
Source: Front Immunol. 2024 May 23;15:1302909. doi: 10.3389/fimmu.2024.1302909 (PMC11153720; doi:10.3389/fimmu.2024.1302909)
Supplement: Supplementary file 1 [file DataSheet_1.docx]

Supplementary Material

# Supplementary Figures


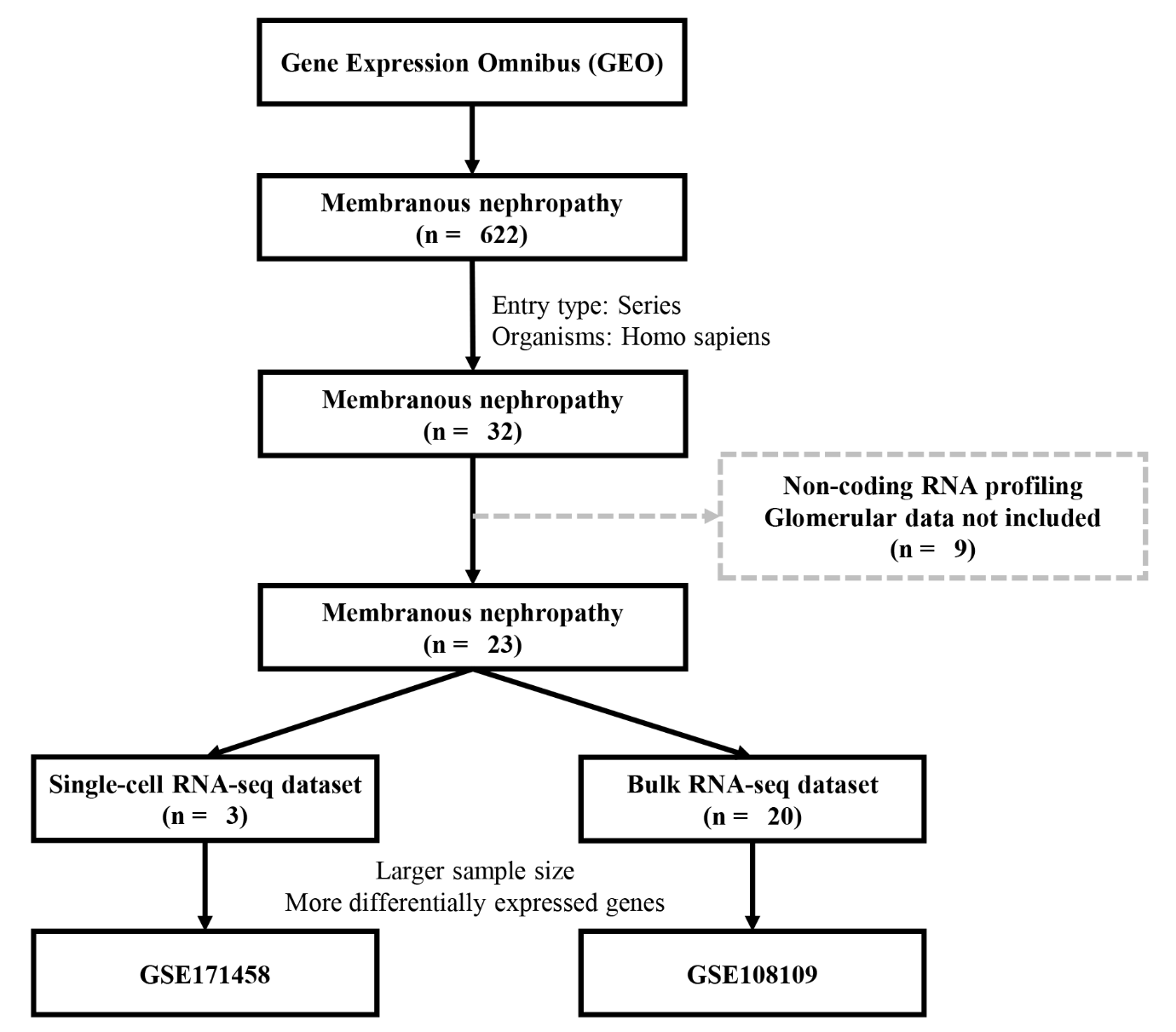


**Supplementary Figure 1.** Search flowchart


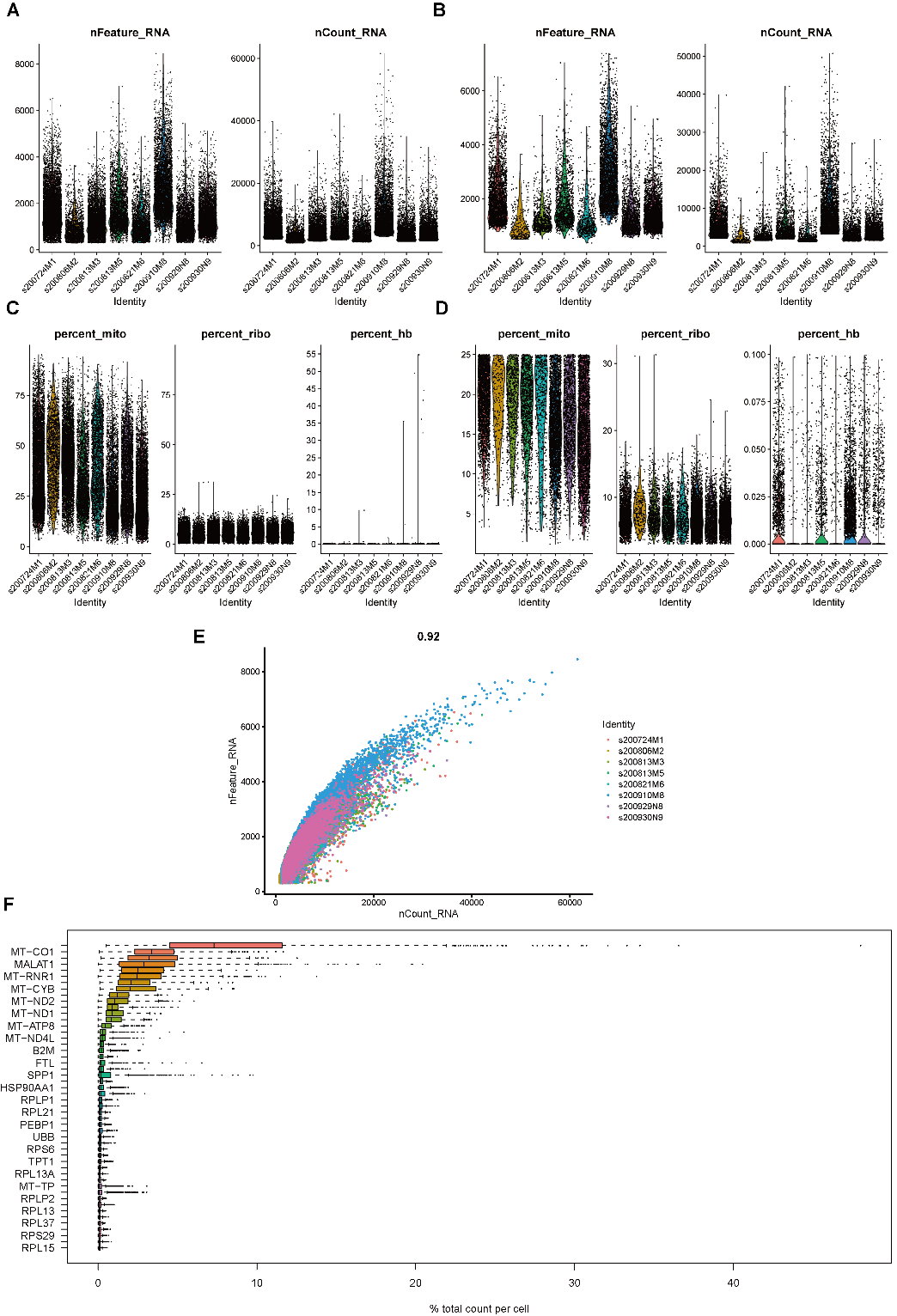


**Supplementary Figure 2.** Essential details of single-cell RNA-seq data pre- and post-filtration. A and B: Number of genes (nFeature_RNA) and transcripts (nCount_RNA) detected in each cell before and after filtration. C and D: Proportions of intracellular mitochondrial genes (percent.mt), ribosomal genes (percent_ribo), and hemoglobin genes (percent_hb) in comparison to overall gene expression before and after filtration. E: Positive correlation observed between nFeature_RNA and nCount_RNA, indicating a relationship between the two. F: Top 25 genes exhibiting high prevalence across cells.


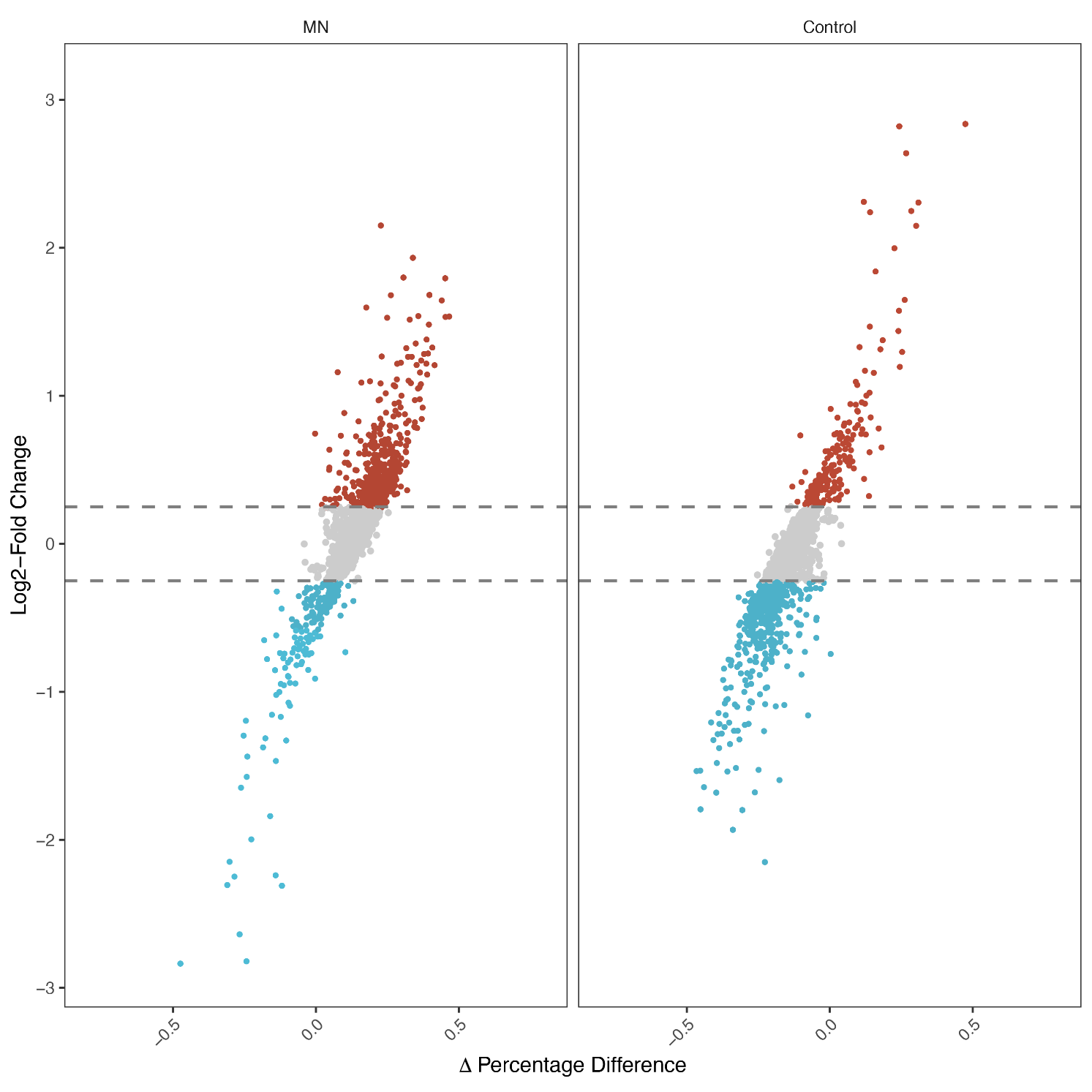


**Supplementary figure 3.** Gene expression levels in the membranous nephropathy group and control group. MN: membranous nephropathy.


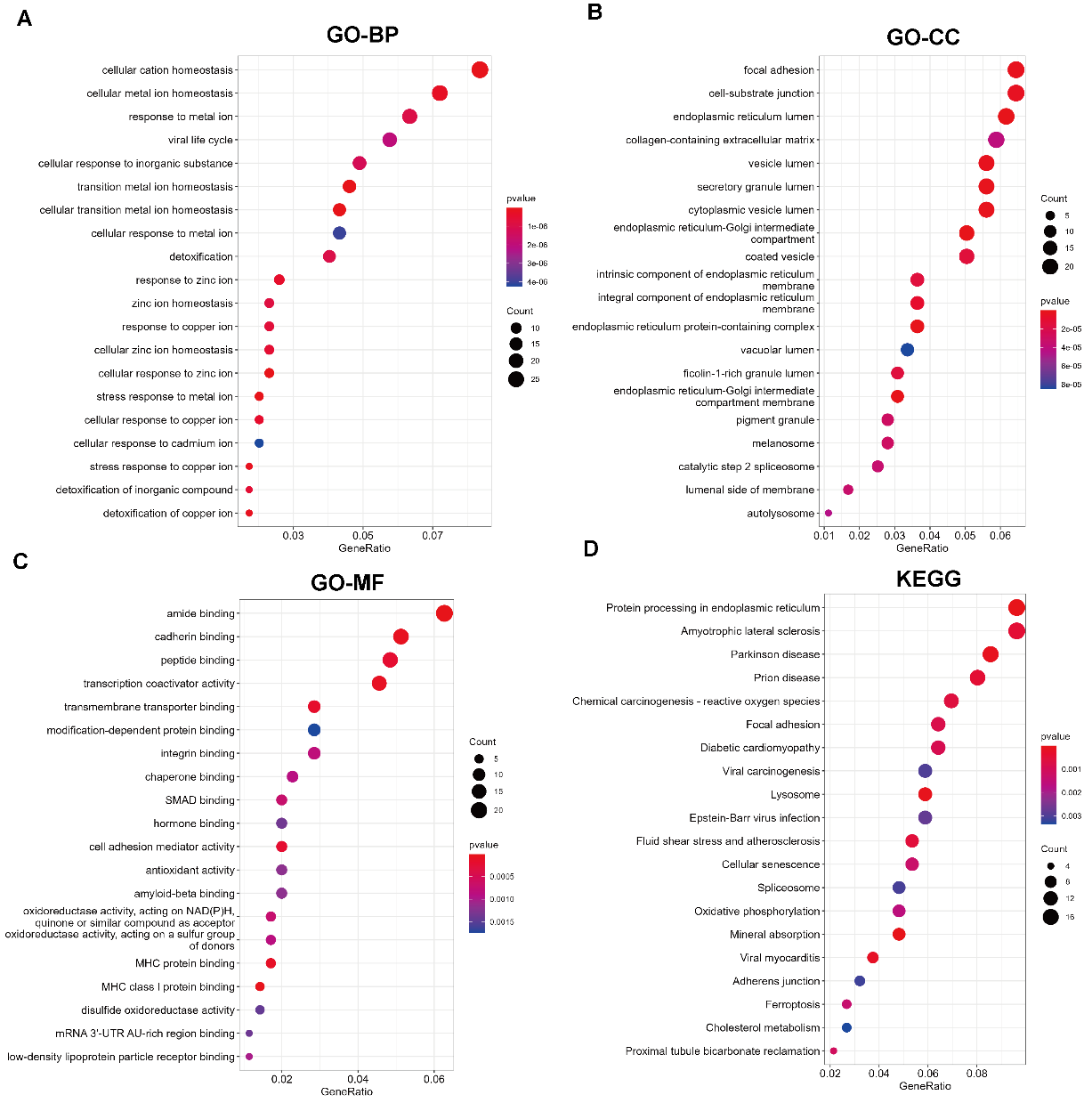


**Supplementary figure 4.** Enrichment analyses with common differentially expressed genes. A-C: GO analysis; D: KEGG pathway enrichment analysis.


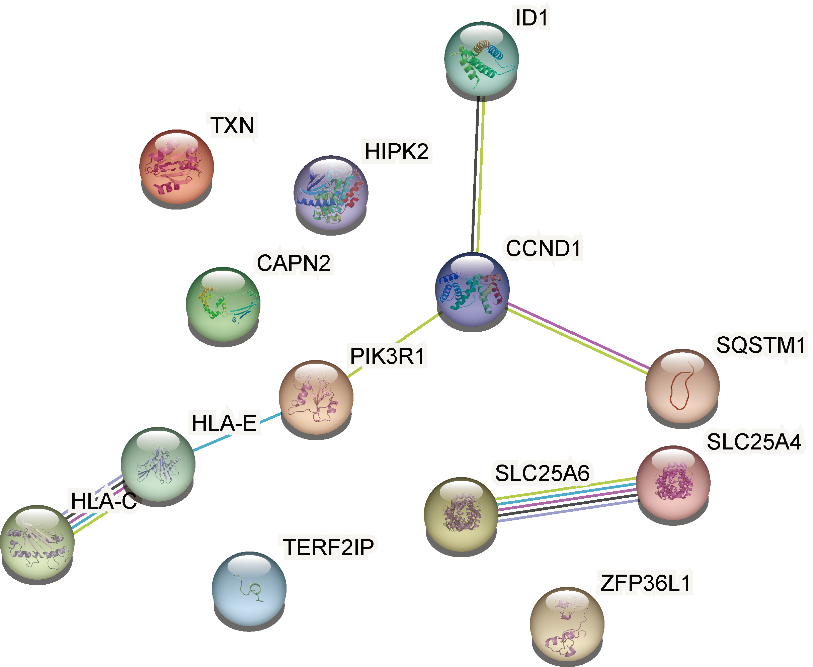


**Supplementary figure 5.** Protein-protein interaction networks. In this network, proteins are represented as nodes, while connecting lines symbolize predicted relationships. Light blue lines indicate auxiliary database evidence, purple lines denote laboratory evidence, yellow lines signify text mining evidence, green lines represent gene similarity, red lines indicate gene fusion, blue lines depict gene co-production, and black lines indicate gene co-expression. Additionally, gray lines signify protein homology.


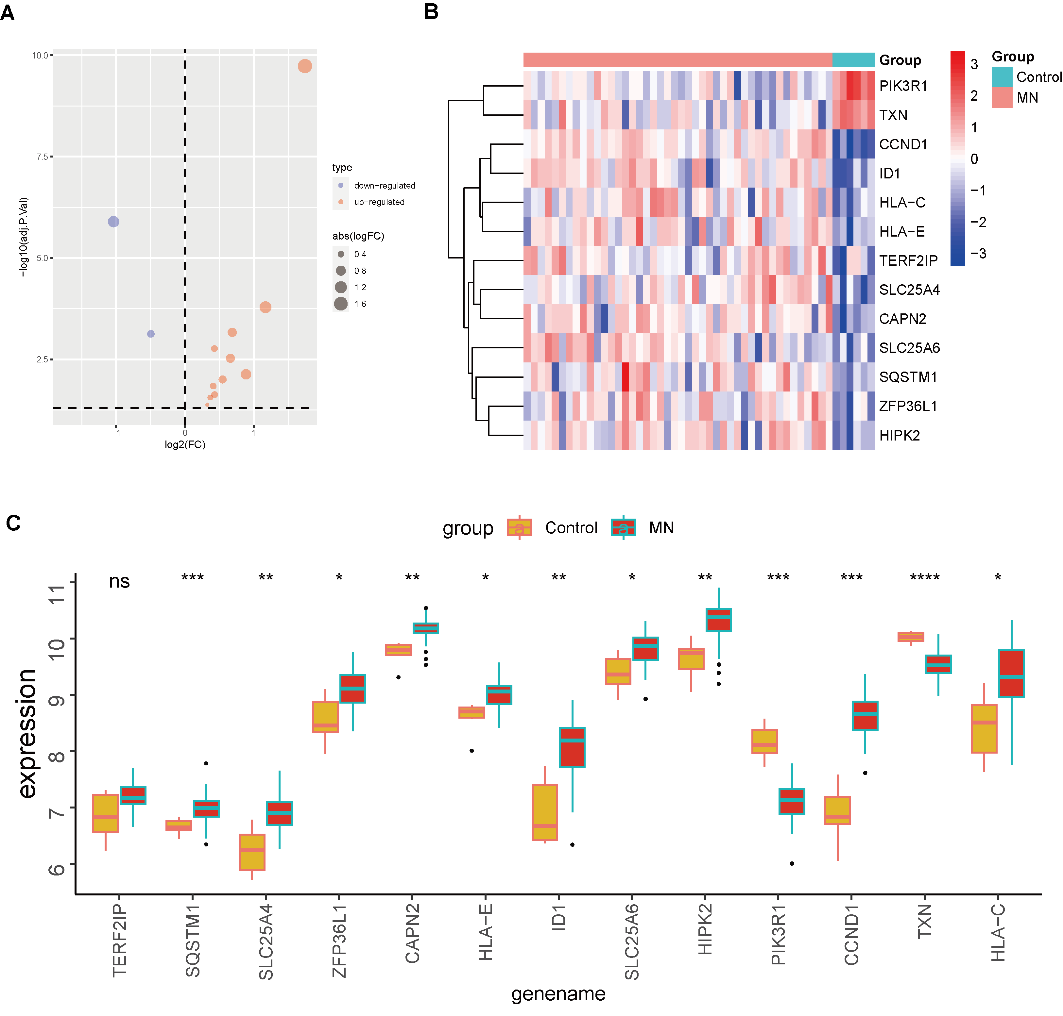


**Supplementary Figure 6.** Expression of senescence-related differentially expressed genes in GSE108109. A: Volcano plot; B: heatmap; C: box plot of senescence-related DEGs expression between groups. ns p > 0.05, *p < 0.05, **p<0.01, *** p < 0.001, **** p <0.0001.


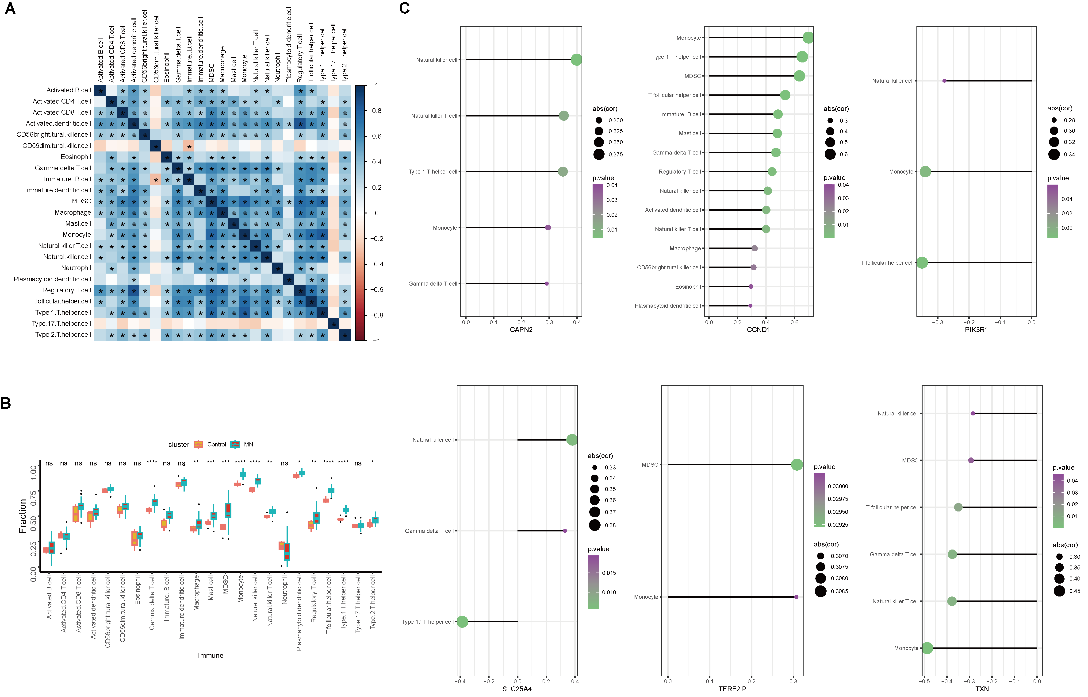


**Supplementary Figure 7.** Immune Infiltration. A: Correlation between immune infiltration cells, where red indicates a positive correlation and blue indicates negative correlation, ∗p < 0.05. B: Expression levels of immune infiltration cells between the MN and control groups, with ns (not significant) when p > 0.05, *p < 0.05, **p < 0.01, ***p < 0.001, ****p < 0.0001. C: Correlation of hub genes with immune infiltration cells, displaying only immune cells with p < 0.05. MDSC: myeloid-derived suppressor cell.


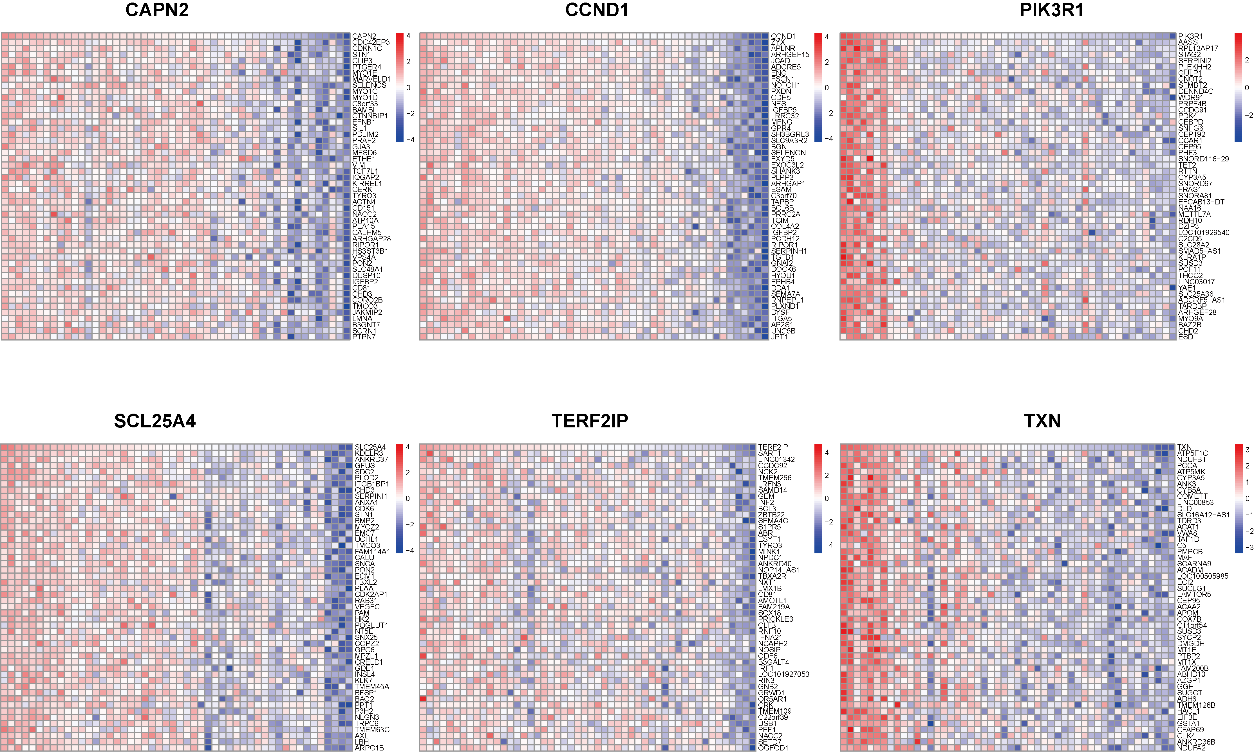


**Supplementary Figure 8.** Correlation Analysis of Hub Genes with All Genes in the Dataset. Only the expression levels of the top 50 positively correlated genes are displayed, with red indicating high expression and blue indicating low expression.


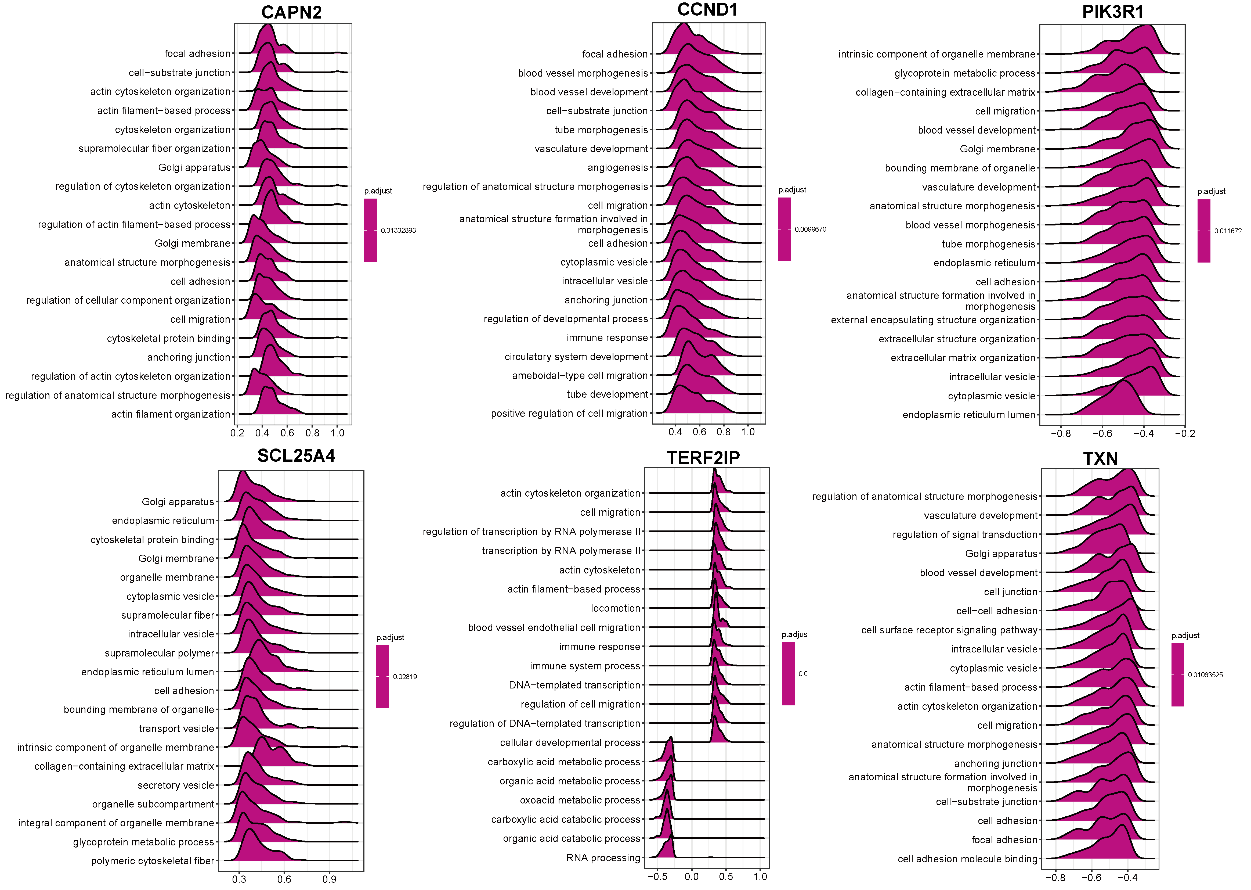


**Supplementary Figure 9.** Gene set enrichment analysis of hub genes. Only the top 20 pathways of single-gene Reactome-based GSEA are shown.
